# A method for the process of collagen modified polyester from fish scales waste

**DOI:** 10.1016/j.mex.2022.101636

**Published:** 2022-02-16

**Authors:** Erh-Jen Hou, Chi-Shih Huang, Ying-Chou Lee, Yu-San Han, Hsueh-Ting Chu

**Affiliations:** a College of Life Science, Institute of Fisheries Science, National Taiwan University, Taipei, Taiwan; b Department of Computer Science and Information Engineering, Asia University, Taichung, Taiwan

**Keywords:** Circular economy, Sustainable aquaculture, Upcycling of fish scale, Collagen modification polyester, Waste management, Functional textiles, UMORFIL, Aquaculture waste

## Abstract

In this study, we introduced a novel polymerization method of polyester using collagen peptides derived from fish scale waste. After the extraction process of collagen peptide from fish scales, putting collagen peptide, ethylene glycol and Benzenedicarboxylic acid into a container, and mixing them to form a mixture; heating the mixture for executing an esterification reaction, to product esters and water; heating the esters, and stirring the esters via a mixer; in a specific period, decreasing the pressure in the container for executing a polycondensation reaction; decreasing the pressure in the container to a second pressure, and stirring the esters via the mixer, to produce a collagen modified polyester.

Collagen peptides are rich in glycine, proline, and hydroxyproline, and by forming a triple helix structure, such as that of the copolyester, gain better hydrophilicity, antistaticity, and ductility. As a result, the produced collagen modified polyester fiber keeps the characteristics of the traditional polyethylene terephthalate fibers including strength, durability, and resistance to wrinkle and shrink. However, the supramolecular collagen modified polyester containing animal collagen peptides has naturally a soft touch and champagne-like color. Consequently, it can be used as a suitable material for skin-friendly functional clothes with or without additional dying. In brief,

• This study introduces a novel method for collagen modified polyester.
• Upcycled fish scale waste brings the sustainable benefits of circular economy.
• Collagen modified polyester provides a new direction for future technological development in the textile industry.

## Graphical abstract

**Figure:**
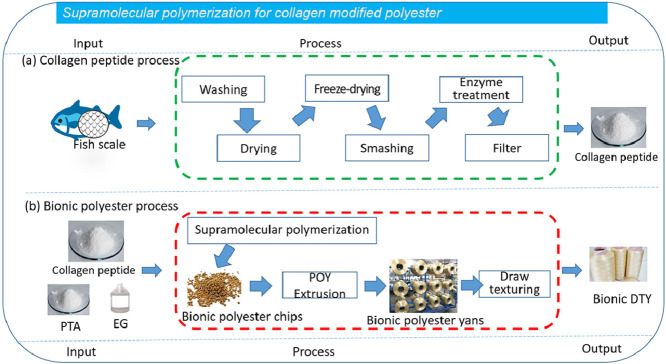

Specifications table

**Table:** 

Subject Area:	Materials Science
More specific subject area:	Textile Material
Method name:	Supramolecular polymerization for collagen modified polyester
Name and reference of original method:	NA
Resource availability:	NA

## Background

Polyester commonly refers to polyethylene terephthalate (PET) whose forming substance is any long-chain synthetic polymer composed of at least 85% by weight of an ester, dihydric alcohol and terephthalic acid. The advantages of polyester include durable, lightweight and anti-wrinkle. The disadvantages of polyester include airtightness and a harder touch. The hygroscopicity of polyester fiber is so poor that the clothing made from polyester fiber makes the skin sticky, clammy and uncomfortable. As a result, polyester fibers and various textile fibers are usually blended or interwoven products, which compensate for the shortcomings of pure polyester fiber fabrics. For example, polyester and cotton blends are common in polo shirts. In addition, polyester and rayon blends are common in men and women suits. Blended or interwoven polyester products are usually more skin-friendly. Besides, application of moisture management chemicals is used to improve the comfort and esthetic properties of polyester fibers [1]. Alkaline and enzyme hydrolysis, plasma, and grafting are popular methods for modifying the chemical and physical characteristics of polyester fabrics for improving their moisture management property [2].

In the food industry, collagen is widely used as a nutritional supplement in the food industry. Recently, due to the mad cow and other problems, collagen extracted from animals has gradually been replaced by other sources [3]. One of the alternative collagen sources is aquaculture waste material (fish skin and scales) [4], [5], [6], [7]. Compared to collagen from animals, collagen from fish scales has advantages such as fat-free, antibiotic-free and prion-free [7]. Consumers often take collagen in nutritional supplements. The nutritional value of collagen peptides from fish scales has also been widely studied [8,9]. Rather than using collagen from scales as a food source, we conceived its application as textile material. Collagens are composed of three α-chains that assemble into complex hierarchical fibers or other structures [10]. We developed a method of producing collagen modified polyester with fish scale-derived collagen in order to improve the properties of common polyester fibers [11].

## The extraction process of collagen peptide from fish scales

**Fig. 1:**
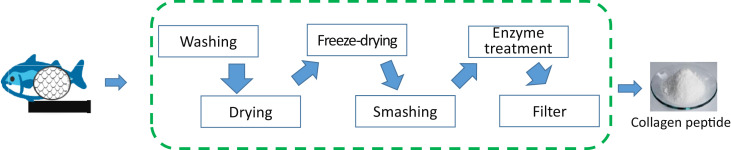
The process of Collagen peptide from fish scales.

The methods to extract collagen peptides from fish skins and scales have been investigated [6,7,12] Fig. 1. Illustrates the process of producing collagen peptide from fish scales [13].

Step 1 (Washing): Wash fish scales with purified water.

Step 2 (Drying): Dry fish scales with cooling fans.

Step 3 (Freeze-drying): Send fish scales into a cold dried chamber. Quick freeze under vacuum conditions.

Step 4 (Smashing): Micronize fish scales by mechanical crusher

Step 5 (Enzyme treatment): Add enzyme to the finely crushed fish scale material, and perform enzyme treatment under warm water.

Step 6 (Filter): After centrifugal filtration of the hydrolyzed liquid, it is dried into a powder.

## Process of producing collagen modified polyester materials

The materials for producing the proposed collagen modified polyester include collagen peptides derived from fish scales, benzenedicarboxylic acid, ethylene glycol and catalysts. The molar ratio of collagen peptides and Benzenedicarboxylic acid was (0.47–0.60):(1.14–1.26). The benzenedicarboxylic acid was terephthalic Acid (TPA) or iso-phthalic Acid (IPA), or a combination of TPA and IPA. The catalysts were Sb_2_O_3_ and TiO_2_ and the ratio range of ppm concentration between Sb_2_O_3_ and TiO_2_ was (160–360):(0–60).

**Fig. 2:**
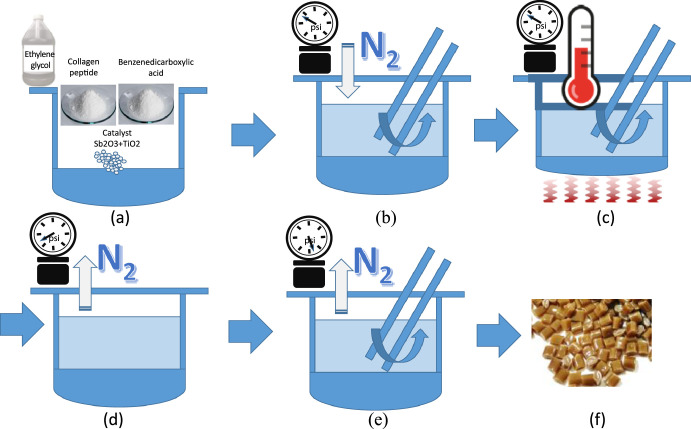
The stages of supramolecular polymerization. (a) mixing of raw materials and catalysts, (b) Introducing nitrogen into the container and stirring the mixture, (c) Heating the mixture for executing an esterification reaction from 210 °C to 270 °C, (d) Decreasing the pressure to 20 torr in the container for a polycondensation reaction, (e) Decreasing the pressure to 3 torr in the container and forming copolyester material and (f) the produced bionic polyester pellets. The values of pressure are not limited thereto and can be changed in accordance with the needs.

**Fig. 3:**
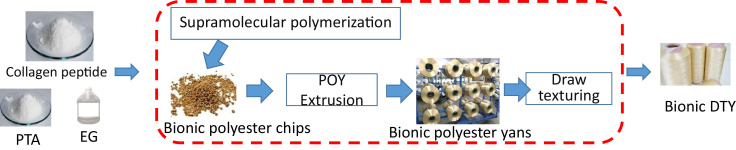
The process of producing collagen modified polyester materials.

The entire process of producing collagen modified polyester is illustrated in Fig. 3 including the three steps:

Step 1 (Supramolecular polymerization): The proposed supramolecular polymerization is an adjusted procedure of normal polymerization method (Fig. 2).

Step 2 (POY Extrusion): The extrusion process includes the melting of polyester chips to form a syrup-like solution which is put in a spinneret and forced through its tiny holes to produce POY (Partially oriented yarns).

Step 3 (Draw texturing): The draw texturing process makes collagen modified silk POY become the collagen modified silk DTY (Draw textured yarns).

## Conclusion

In summary, we introduced a new process for producing collagen-modified polyester from fish scale waste. This produced material has excellent deodorant and moisture regain activity, and is friendly to the skin. The collagen extracted from fish scales helps to reuse aquaculture waste. The manufacturing process changes the molecular structure of the polyester, and gives it a natural champagne gold color. The produced collagen modified polyester material can be used in many products such as base fabrics, socks, tops, denim fabrics, outerwear, bedding, etc.

## References

[1] Ali A. (2017). Effect of jute fibre treatment on moisture regain and mechanical performance of composite materials. IOP Conf. Ser. Mater. Sci. Eng..

[2] Chowdhury M., Alam M. (2017). Improvement of moisture management of polyester fabric using moisture management chemical. Int. J. Appl. Manag. Sci..

[3] Mahboob S. (2015). Isolation and characterization of collagen from fish waste material- skin, scales and fins of Catla catla and Cirrhinus Mrigala. J. Food Sci. Technol..

[4] Dauda A.B., Ajadi A., Tola-Fabunmi A.S., Akinwole A.O. (2019). Waste production in aquaculture: sources, components and managements in different culture systems. Aquac. Fish..

[5] Harikrishna N., Mahalakshmi S., Kumar K.Kiran, Reddy G. (2017). Fish scales as potential substrate for production of alkaline protease and amino acid rich aqua hydrolyzate by bacillus altitudinis GVC11. Indian J. Microbiol..

[6] Kumar B., Rani S. (Jan 2017). Technical note on the isolation and characterization of collagen from fish waste material. J. Food Sci. Technol..

[7] Chinh N.T. (2019). Characterization of collagen derived from tropical freshwater carp fish scale wastes and its amino acid sequence. Nat. Prod. Commun..

[8] Paul C., Leser S., Oesser S. (2019). Significant amounts of functional collagen peptides can be incorporated in the diet while maintaining indispensable amino acid balance. Nutrients.

[9] Zdzieblik D., Oesser S., Baumstark M.W., Gollhofer A., König D. (2015). Collagen peptide supplementation in combination with resistance training improves body composition and increases muscle strength in elderly sarcopenic men: a randomised controlled trial. Br. J. Nutr..

[10] Saxena T., Karumbaiah L., Valmikinathan C.M., Kumbar S.G., Laurencin C.T., Deng M. (2014). Natural and Synthetic Biomedical Polymers.

[11] Hou E.J., Huang C.S., Lee Y.C., Chu H.T. (2021). Upcycled aquaculture waste as textile ingredient for promoting circular economy. Sustain. Mater. Technol..

[12] Ennaas N. (2016). Collagencin, an antibacterial peptide from fish collagen: activity, structure and interaction dynamics with membrane. Biochem. Biophys. Res. Commun..

[13] Suo-Lian W., Huai-Bin K., Dong-Jiao L. (2017). Technology for extracting effective components from fish scale. J. Food Sci. Eng..

